# A Synopsis of Biomarkers in Glioblastoma: Past and Present

**DOI:** 10.3390/cimb46070412

**Published:** 2024-07-03

**Authors:** Ligia Gabriela Tataranu, Serban Turliuc, Radu Eugen Rizea, Anica Dricu, Oana Alexandru, Georgiana-Adeline Staicu, Amira Kamel

**Affiliations:** 1 Neurosurgical Department, University of Medicine and Pharmacy “Carol Davila”, 020022 Bucharest, Romania; rizea.radu.eugen@gmail.com; 2 Neurosurgical Department, Clinical Emergency Hospital “Bagdasar-Arseni”, 041915 Bucharest, Romania; kamel.amyra@yahoo.com; 3 Medical Department, University of Medicine and Pharmacy “G. T. Popa”, 700115 Iasi, Romania; serban_turliuc@yahoo.com; 4 Department of Biochemistry, Faculty of Medicine, University of Medicine and Pharmacy, 200349 Craiova, Romaniaoanale@hotmail.com (O.A.); adstaicu@gmail.com (G.-A.S.)

**Keywords:** glioblastoma, biomarkers, immunotherapy, biomolecular, neurosurgery

## Abstract

Accounting for 48% of malignant brain tumors in adults, glioblastoma has been of great interest in the last decades, especially in the biomolecular and neurosurgical fields, due to its incurable nature and notable neurological morbidity. The major advancements in neurosurgical technologies have positively influenced the extent of safe tumoral resection, while the latest progress in the biomolecular field of GBM has uncovered new potential therapeutical targets. Although GBM currently has no curative therapy, recent progress has been made in the management of this disease, both from surgical and molecular perspectives. The main current therapeutic approach is multimodal and consists of neurosurgical intervention, radiotherapy, and chemotherapy, mostly with temozolomide. Although most patients will develop treatment resistance and tumor recurrence after surgical removal, biomolecular advancements regarding GBM have contributed to a better understanding of this pathology and its therapeutic management. Over the past few decades, specific biomarkers have been discovered that have helped predict prognosis and treatment responses and contributed to improvements in survival rates.

## 1. Introduction

The worldwide incidence of brain cancer in 2022 has been reported to be 3.5%, with a 5-year prevalence of 2.2 per 100.000 cases [1]. In Europe, a higher percentage of new cases was reported, 5.6%, with a 5-year prevalence of 2.0 per 100.000 cases [2], while in Romania, there have been 6.5% newly reported cases of brain malignancies, with a 0.56 cumulative risk and a 5-year prevalence of 2.7 per 100.000 cases [3].

With a median age of 64 years, the most frequent brain malignancy (48%) in adults is represented by glioblastoma (GBM) [4,5], a tumor with extremely aggressive features and a dismal prognosis [6]. However, a good Karnofsky performance scale (KPS) score and young age have been reported to be positive prognostic factors [7]. The diagnosis is commonly based on neuroimaging and stereotactic tissue biopsy or tumor resection [5].

GBMs are the most frequent primary brain tumors, exhibiting aggressive behavior characterized by invasiveness, resistance to current therapeutical options, and recurrence [5]. Regarding its clinical presentation, GBM used to be categorized as primary and secondary prior to 2021 [8]. Primary, or de novo, GBM accounts for approximately 90% of cases and arises in patients with no previous history of brain tumors, particularly in the elderly [9]. This type of GBM typically evolves from glial cells [9]. On the other hand, secondary GBM evolves from low-grade tumors, mainly in younger patients [9]. However, according to the World Health Organization (WHO) 2021 Classification of Central Nervous System Tumors, fifth edition (CNS5), GBM is no longer described as primary and secondary. Regarding the taxonomy, the term “entity” has been replaced by “type”, and the term “variant” has been replaced by “subtype” [10]. Although previously the tumor grading was made based on histopathological characteristics, the new classification concluded that “molecular beats histology” because molecular biomarkers can more accurately predict the prognosis and recurrence of a disease [10]. Moreover, the authors suggest that CNS tumor classification will soon be based solely on molecular genetic analyses [10]. Based on the latest classification, adult-type diffuse gliomas represent a tumor group that includes astrocytoma IDH-mutant; oligodendroglioma IDH-mutant and 1p/19q-codeleted; and glioblastoma, IDH-wild-type [11]. Typically, GBM IDH-wild-type CNS WHO grade 4 exhibits necrosis and microvascular proliferation. Given the fact that IDH-wild-type astrocytomas have similar behavior to GBM, WHO CNS5 defined as GBM IDH-wild-type CNS WHO grade 4 any IDH-wild-type astrocytomas with at least one of the following molecular characteristics: EGFR amplification, TERT promoter mutations, or gain of chromosome 7 and loss of chromosome 10 [12]. Nonetheless, it has been stated that an astrocytoma IDH-wild-type in the absence of the histopathological and molecular characteristics of GBM is a very rare type [13].

The update on nomenclature aims to facilitate the diagnosis, treatment, and prognosis of CNS tumors. Thus, according to WHO CNS5, the 2016 secondary GBM becomes astrocytoma IDH-mutant CNS WHO grade 4 [10]. The diffuse astrocytoma, IDH-wild-type, WHO grade II, as well as the anaplastic astrocytoma IDH-wild-type WHO grade III, will both become glioblastoma IDH-wild-type CNS WHO grade 4 if they feature TERT promoter mutation, EGFR amplification, and/or +7/−10) [10]. Finally, primary GBM will become glioblastoma IDH-wild-type CNS WHO grade 4 as well [10].

Although GBM mostly arises in the brain hemispheres, cases of various locations have been reported, such as the brainstem [14], cerebellum [15], and spinal cord [16,17].

While there is currently no curative treatment for GBM, many pharmacological and biomolecular interventions have revealed alterations in the course of the disease [6,7]. Particular tumoral vulnerabilities, such as mesenchymal–epithelial transition (MET) gene amplification or fusions [18,19], fibroblast growth factor receptor (FGFR) gene fusions [20], or v-Raf murine sarcoma viral oncogene homolog B (BRAF) mutation [21], may be susceptible to targeted interventions [7]. Currently, the main therapeutic approach in GBM is multimodal and consists of neurosurgical intervention, radiotherapy, and chemotherapy [22]. Although image-guided radiation therapy (IGRT) is not standard practice now, classical radiotherapy might slowly be replaced by tomotherapy, which has demonstrated superior results, particularly in terms of preserving healthy tissue, due to its increased precision and safety [23,24]. It is worth noting that a major obstacle to radiotherapeutic effectiveness is represented by the hypoxic tumoral microenvironment. This type of hypoxic tumoral tissue requires a higher dose of radiation [25,26].

The standard and most-used chemotherapeutic agent in GBM is temozolomide (TMZ) [27]. The vast majority of patients will eventually develop resistance to monotherapy, but when combined with different immunotherapies, TMZ may have unpredicted results [27,28,29]. Despite the increased availability of novel therapies, neurosurgical intervention still represents the main approach in symptomatic patients with GBM, providing the major advantage of the quick reversal of neurological manifestations [30,31]. The extent of resection has a key role in surgical management, as it can predict long-term survival and even clinical trial eligibility [30]. Even though maximal safe resection is recommended, the infiltrative nature of this type of tumor makes it strenuous [30]. Although most patients will develop treatment resistance and tumor recurrence after surgical removal, biomolecular advancements regarding GBM have potentially contributed to a better understanding of this pathology and its therapeutic management. Over the past decades, specific biomarkers have been discovered and associated with GBM, predicting the response to treatment and contributing to the improvement of survival rates [32,33].

### Tumor Microenvironment

For a tumor to develop and grow, numerous cellular and noncellular components are required, and these comprise the tumoral microenvironment (TME) [34]. The key elements of the TME are represented by the extracellular matrix (ECM), blood vessels and lymphatic networks, immune and inflammatory cells, and fibroblasts. In GBM, the TME can secrete many factors, and, in addition to the aforementioned TME elements, various signaling pathways have been described [34]. However, it is worth mentioning that the TME can be impacted by GBM’s molecular patterns [35].

In order to develop new therapeutic strategies for GBM, it is vital to understand the mechanisms of cellular communication between the TME and glioma stem cells, as numerous malignant processes are supported by cell–TME–cell interactions [35].

The GBM ECM has a different composition to the healthy ECM, with higher concentrations of hyaluronan, collagen, glypican-1, neurocan, NG2 proteoglycan, versican, and tenascin-C [36]. Additionally, CD147 is produced, which is a key factor in the process of tumoral growth [36]. Another important aspect regarding the EMC in GBM is represented by a special subtype of microenvironment around neural stem cells, also known as a stem-cell niche, which is responsible for tumoral development and progression, as well as treatment resistance and relapses [36].

The blood–brain barrier (BBB) in GBM is compromised due to inflammation, compression, and tumoral neovascularization (mainly on account of vascular endothelial growth factor in high amounts), which in the end leads to high GBM perfusion. The compromised BBB is also responsible for hypoxia and the presence of macrophages, which promote immunosuppression and GBM invasion by the CCL4-CCR5 axis [37,38,39].

In GBM, immune cells can make up approximately 50% of the tumoral mass, especially myeloid cells, which are represented by microglia, neutrophils, dendritic cells, bone marrow-derived macrophages, and myeloid-derived suppressor cells [40]. The microglia are mainly on the tumoral edges and have no suppressive function. Neutrophils, myeloid-derived suppressor cells, and bone marrow-derived macrophages are correlated with a dismal prognosis, a poor survival rate, and a higher rate of recurrence. Dendritic cells are correlated to anti-tumoral responses and T cell infiltration [40]. A crucial cytokine contributing to the TME is transforming growth factor beta, which in GBM is secreted in high levels and contributes to neovascularization and immune evasion. In the future, this could be considered as a potential therapeutic target [41].

Cancer-associated fibroblasts are a major element of tumoral stroma in cancerous lesions. However, in the brain, the role of astrocytes overlaps with that of the fibroblasts in other parts of the body; thus, they have a similar function in tumoral growth. Moreover, in GBM, fibroblast activation protein could be a major target for therapy [42].

Regarding signaling molecules in GBM, various cytokines and chemokines are produced in the TME by inflammatory pathways. The activation of NF-kappa B not only promotes tumoral growth and invasiveness but also induces treatment resistance. STAT3 also plays a crucial role in the GBM TME by inducing tumoral growth and invasiveness, sustaining neovascularization, and preventing cellular apoptosis [42].

In GBM, the TME is an important topic, given the fact that it can be a great anti-cancer therapeutic target by providing numerous biomarkers that will be discussed further [42].

## 2. Immunotherapies and the Need for Biomarkers in GBM [43]

Considering the complexity of genomics and the numerous alterations in signaling pathways in GBM, continuous efforts have been made to implement new systemic therapies and improve existing ones. Therefore, new therapeutic strategies for GBM emerged [33]. Immunotherapeutic agents harness the human immune system in order to destroy cancerous cells [44]. Major advances have been made in cerebral malignancies, with reported cases of complete tumoral remission after the administration of systemic monotherapy [45]. However, although immunotherapeutic agents might improve outcomes in the management of other diseases, GBM patients may not substantially benefit from them, [46] or might benefit insufficiently [47]. These results explain the high demand for further effective therapeutic research. This main goal leads to the creation of accurate clinical measurement tools to assess disease progression and the results of any therapeutical intervention [43]. This is how biological markers (biomarkers) from many categories have been created. Thus, biomarkers are analytical instruments that can measure biological parameters, describing empirical results. These tools can be obtained from molecular, histologic, or physiologic materials [48]. Currently, GBM biomarkers can predict the origin, diagnostic features, progression, and response to treatment [11]. Although biomarkers can also be important tools to evaluate the safety or efficacy of treatment, some of them can evaluate both simultaneously [13,43]. These biological tools can be generated by the human body as a reaction to a cancerous disease but can also be a direct product of cancerous cells [49].

Biomarkers in GBM have substantially influenced its classification as a consequence of consistently being defined by molecular characteristics [11], as we have already mentioned. A variety of biomarkers have been discovered in GBM, which mainly comprise genetic, molecular, circulating, and antiangiogenic biomarkers. The theory of multimodal biomarkers has been studied lately, as well as the topics of delayed hypersensitivity, reactive oxygen species, hypoxia, and autophagy [6,11]. All these specific biomarkers are going to be discussed further.

## 3. Researched Past and Current Biomarkers in GBM

### 3.1. Genetic Markers in GBM (Table 1)

#### 3.1.1. Isocitrate Dehydrogenase (IDH)—IDH-Mutated versus IDH-Wild-Type Tumors

Isocitrate dehydrogenase is the major source of nicotinamide adenine dinucleotide phosphate (NADP^+^) production in the cytosol of brain cells, and both IDH1 (cytoplasmic isoform) and IDH2 (mitochondrial isoform) reduce the DNA damage and lipid peroxidation [6]. In 2008, the first article to report the expression of IDH mutation in GBM was published by Parsons et al. The authors of the study discovered that IDH1 could be a potential marker for secondary GBM [50], currently known as astrocytoma IDH-mutant CNS WHO grade 4 [10]. While WHO CNS 5 no longer considers IDH as a specific biomarker for GBM, we will provide a concise analysis of IDH-mutated versus IDH-wild-type tumors, particularly in relation to the new classification.

The term IDH-mutant is no longer correlated with GBM; instead, it now comprises all IDH-mutated diffuse astrocytomas, which are graded mainly on histological features [51]. However, the alterations in Cyclin Dependent Kinase Inhibitor 2A (CDKN2A) and/or CDKN2B predict a dismal prognosis in this category, and regardless of any other morphological characteristics, the presence of alterations in CDKN2A/B will result in CNS WHO grade 4 [12,51].

Generally, IDH-mutated tumors are correlated with longer survival rates [52], especially when compared to individuals with IDH-wild-type tumors that do not exhibit IDH mutation [53]. It is worth mentioning that on a genomic basis, IDH-mutated tumors are very distinct from wild-types, although histologically they are similar [54]. In IDH-mutated tumors, a base arginine–histidine substitution at codon 132 of IDH1, called R132H mutation, was preponderant, while in some other tumors with IDH2 mutation, the arginine is replaced by lysine. This had a significant impact on the metabolic activity of isocitrate and therefore affected the epigenetic and metabolic state of tumoral cells [55,56]. Even though the existence of IDH1 and IDH2 mutations are mutually exclusive [56], rare cases of co-occurrence have been recently reported [57]. Usually, the typical function of IDH1 and 2 is to convert isocitrate into alpha-ketoglutarate (AG), which is an intermediate product of the Krebs cycle [58]. However, when mutations occur, AG is converted into 2-hydroxyglutarate (2HG), an indispensable oncometabolite in the formation of gliomas [59]. 2HG has a relatively similar structure to AG, and it is created when the ketone group of AG is reduced to a hydroxyl group [58]. Additionally, given the fact that MRI-spectroscopy can assess the levels of 2HG, it has been demonstrated that this can be a predictor for treatment response [60].

Presently, GBMs are classified as adult-type tumors and are not IDH-mutated anymore but only IDH-wild-type. An IDH-wild-type tumor, H3-wild-type, with necrosis or microvascular proliferation is considered GBM IDH-wild-type, grade 4 (adult-type diffuse glioma) [61]. Also, an IDH-wild-type, H3-wild-type, with TERT promoter mutation, EGFR amplification, or +7/−10 is considered GBM IDH-wild-type, grade 4 (adult-type diffuse glioma) [61]. However, an IDH-wild-type tumor, H3-wild-type, with H3 K27me3 loss, EZHIP overexpression, or EGFR mutation is considered a diffuse glioma, H3 K27-altered, grade 4 (pediatric-type high-grade glioma) [61].

In addition, according to WHO CNS5, patients older than 55 years without immunoreactivity to IDH1 R132H, with the histopathological characteristics of GBM, with other than midline location, and with no history of glioma, will be diagnosed as GBM IDH-wild-type CNS WHO grade 4 [10].

**Table 1:** Summary of described genetic biomarkers in GBM.

Biomarker Name	Identified Trait in GBM	Impact on GBM
**Isocitrate dehydrogenase (IDH)**	Mutation used to differentiate IDH-mutated GBM from IDH-wild-type GBM before WHO CNS 5.	Considered for a very long time as a major diagnostic and prognostic element that predicted survival outcomes and treatment response.
**O6-methylguanine DNA methyltransferase (MGMT)**	The loss of long arm of chromosome 10 (70%). The methylation of MGMT promoter leads to an inactivation of the MGMT gene or a loss of expression.	Predicts treatment responses to alkylating chemotherapy (e.g., temozolomide) and survival outcomes. Predicts treatment responses to chlorethylating agents.
**Phosphatase and TENsin homolog (PTEN)**	Mutations/altered or decreased PTEN. Overexpression/deletion of the tumor suppressor gene PTEN.	Key factor in tumoral growth, cellular proliferation, and invasion. Altered or decreased PTEN expression level correlates with increased disease aggressiveness, worse prognosis, and poor overall survival. Deletion can be related to drug resistance. Overexpression could contribute to DNA damage, promoting radio-sensitivity.
**Loss of heterozygosity (LOH)**	Chromosomal gain/loss/alteration.	Absence of 1p and 19q chromosomal arms was associated with better progression-free survival and a longer overall survival rate. LOH on chromosomes 1p, 9p, 10q, 17p, and 19q was associated with a poor prognosis. Whole chromosome 7 gain increases the probability of recurrence and is associated with shorter survival rates when compared to EGFR amplification. LOH on chromosome 10 can be a useful genetic marker in GBM for prognosis and diagnosis.
**Neurofibromatosis type 1 gene (NF-1)**	Inactivation (due to mutation/deletion/loss)/low expression.	Low expression and loss of function in NF-1 are strongly associated with the development of mesenchymal GBM subtype.

#### 3.1.2. The Epigenetic Modification of O6-Methylguanine DNA Methyltransferase (MGMT)

**Figure 1:**
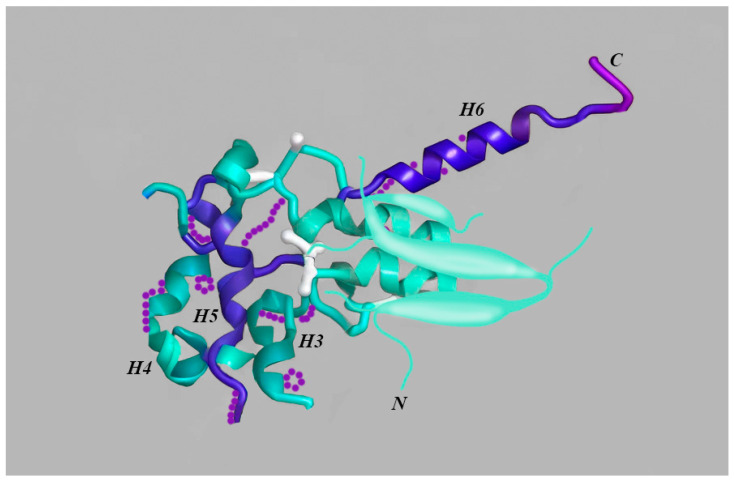
A schematic rendering of MGMT showcasing its complex molecular structure. The carboxyl-terminal tail can shift to take in the DNA substrate and have an important role in substrate specificity. Also, the C-terminal tail has a role in modulating MGMT activity [62,65]. The other helical regions, H3, H4, and H5, comprise the putative DNA binding section. The purple dots represent the side chains of the residues and are familiar to every alkyltransferase. The N-terminal domain has a crucial role in preserving structural stability. Additionally, when two domains are individually expressed, the N-terminal domain can stabilize the formed complex [65].

One of the main cellular roles is the repair of damaged DNA. This function requires a broad spectrum of gene products, and while a certain amount of repair pathways control numerous types of lesions, others are extremely specific [62]. Even though many repair reactions require various proteins and gene products, there is a single protein that can independently repair the DNA in only one step [62]. This protein is referred to as O6-alklylguanine-DNA alkyltransferase (AT or AGT), or O6-methylguanine DNA methyltransferase (MGMT) (Figure 1) [62]. The repair reaction of MGMT is based on the removal of DNA adducts established at the O6 position of guanine and also at the O4 of thymine, since these specific DNA adducts are paramount elements involved in the carcinogenic, toxic, and mutagenic effects of alkylating agents [62]. In GBM, the loss of the long arm of chromosome 10 (10q) is noted in approximately 70% of cases, and according to WHO CNS5, is a main feature for diagnosis [62]. This is particularly important to mention since MGMT is located on chromosome 10q26 [63]. The methylation of MGMT promoter leads to an inactivation of the MGMT gene or a loss of expression. As a result, the double inactivation of MGMT, by both chromosome loss and the hypermethylation of its promoter, leads to chemosensitivity when it comes to alkylating agents such as TMZ [63]. Therefore, the methylation status of this biomarker has a remarkable clinical importance [64].

It has been concluded that when compared to individuals without MGMT methylation, those with MGMT methylation may have a longer overall survival, both when treated with alkylated agents alone or associated with tyrosine kinase inhibitors or immunotherapy (HR ratio of 1.64; 95% CI 1.23–2.18; *p* = 0.0007) [33,63,66]. It has also been shown that there is a correlation between the status of MGMT promoter methylation and disease prognosis when treated with TMZ [67]. In older people, survival rates were longer in methylated individuals treated with TMZ. However, radiotherapy in monotherapy had better results in unmethylated patients [68].

A notable variability was observed in the overall survival rates among patients treated with bischloroethylnitrosourea (BCNU) based on MGMT levels in tumoral specimens. The concluded differences indicate that MGMT can be used as a biomarker to predict the treatment response to chloroethylating agents [67].

When it comes to the regulation of MGMT, understanding the process is of major significance for developing new targeted agents or improving existing ones. Worth mentioning is the existence of other genes associated with MGMT expression in GBM. For example, the expression of MGMT is negatively regulated by p53, and lower MGMT expression is correlated with better outcomes with specific phenotypes that express p53 [69]. Therefore, the reactivation of p53 could be a step towards overcoming GBM’s resistance to TMZ and tumoral growth [69].

Nevertheless, GBM patients will relapse almost every time, with GBM typically becoming more aggressive and more resistant to therapy [70]; neurosurgical intervention followed by TMZ treatment is frequently a feasible option [71]. Consequently, the methylation of MGMT promoter can be predictive of the outcome in GBM recurrences [71].

In conclusion, when it comes to DNA methylation, one of the main markers in GBM is represented by MGMT promoter. While this epigenetic biomarker is correlated with the transcriptional silencing of the MGMT gene, it has been stated that in GBM it can be used in predicting treatment responses [63]. The theranostic hallmark of GBM is represented by the combination of the loss of the long arm of chromosome 10 and the hypermethylation of MGMT, highlighting individual cases in which TMZ therapy could be beneficial [63].

#### 3.1.3. Phosphatase and TENsin Homolog (PTEN)

The PTEN gene is a tumor suppressor gene located on chromosome 10q23.3, and it was first described in 1997 [72]. The PTEN protein plays a crucial role in cell growth regulation, proliferation, and survival, being involved in the molecular pathogenesis of gliomas [73]. The tumor suppression feature of PTEN is related to its lipid phosphatase activity, which leads to the inhibition of PI3K/Akt signaling [73]. Generally, the PI3K/Akt pathway exhibits oncogenic activity. During tumor growth, this pathway is overactivated [74]. When PTEN is mutated, it affects the PI3K/Akt/mTOR pathway [74], leading to uncontrolled cell proliferation and tumor cell invasion by blocking apoptosis [75]. Some studies have shown that PTEN also has an individual role in gliogenesis [74], while being the most common mutation in GBM [76]. Altered or decreased PTEN expression levels correlate with increased disease aggressiveness, worse prognosis, and poor overall survival [72]. Moreover, the deletion of the tumor suppressor gene PTEN and the induction of PI3K/Akt signaling can be correlated with drug resistance [74,77]. PTEN overexpression could contribute to DNA damage, promoting radio-sensitivity [74].

Considering the importance of PTEN in the pathogenesis of glioblastoma, anti-cancer agents targeting PTEN signaling in glioblastoma were studied. Among them, curcumin, tangeretin, and silibinin showed increased PTEN expression and downregulation of the PI3K/Akt pathway [74]. Other anti-cancer agents, such as celastrol, taxifolin, and punicic acid, target the PI3K/Akt/mTOR pathway and have the ability to inhibit this axis, reducing the malignancy of GBM cells and also decreasing the chance of resistance to therapy [74].

#### 3.1.4. Loss of Heterozygosity (LOH)

The loss of heterozygosity represents a very frequent event in various malignancies, and it is characterized by the somatic loss of genetic material from one allele of specific genes [78]. The etymology of the word refers to a marker modification from a heterozygous form to a homozygous one, a change that might appear in the course of the tumor formation [79]. Although most of these genetical events are a result of a loss of allele due to a reduction of gene copies, cases of allelic loss with a constant number of gene copies or even an increased number have often been described [80]. In the first case, a chromosomal component is deleted, and sometimes the entire chromosome, while in the second case, a gene conversion or a chromosomal duplication is observed [78]. Studies regarding chromosomal loss or gain in GBM had a major impact on GBM diagnosis, and currently, they are the basis of WHO CNS5 [51].

The main genetic alteration (up to 80% of cases) in GBM is represented by LOH on chromosome 10, and in approximately 20% of cases, the PTEN found on 10q23.2 was mutated [11,81]. Due to the altering of PTEN, the defense mechanisms against tumor formation are affected [78]. PTEN alterations will support tumoral growth, while the loss of PTEN 10q25-qter loci will impact the evolution to GBM [81] and will have utility in the diagnosis of GBM [11,82]. LOH on chromosome 10 can be a useful genetic marker in GBM, as patients with this alteration have shorter survival rates in comparison to patients who do not have it [82]. Furthermore, the MGMT gene is positioned on 10q26 [83], and recent research concluded that patients with LOH on 10q and hypermethylation of MGMT promoter have longer survival rates (21.5 months versus 8.1 and 9.5 months in other groups) and better therapeutic responses to chemotherapy with TMZ [63].

In GBM, the absence of both 1p and 19q chromosomal arms was associated with better progression-free survival and a longer overall survival rate [84]. However, if analyzed separately, the results were different. Jesionek-Kupnicka and colleagues analyzed the LOH on chromosomes 1p, 9p, 10q, 17p, and 19q in GBM patients and concluded that patients with LOH in at least one of these loci had remarkably poor outcomes when compared to patients without LOH [85]. The survival rates of the first group ranged from 2–19 months, while in patients without LOH, it ranged from 6–48 months. Similar results were reported by Huang et al. regarding the LOH of 9p [86].

Whole chromosome 7 gain in GBM was found to increase the probability of recurrence and was associated with shorter survival rates when compared to EGFR amplification [87]. In addition, chromosome 7p or 7q gain and loss of chromosome 10p or 10q were associated with tumoral aggressiveness and shorter survival rates in individuals with WHO grade II or III IDH-wild-type astrocytic gliomas [12].

Nevertheless, the gain of chromosome 7/loss of chromosome 10 has great diagnostic specificity. In rare cases, supplementary tests might be needed, such as BRAF V600E testing [88]. Although other imbalances such as +7q/−10q, +7/−10q, or +7q/−10 have been reported, the dominant signature remains the gain of whole chromosome 7 and the loss of whole chromosome 10, which can currently be used to diagnose IDH-wild-type GBM [11,12].

LOH in at least one locus on the long arm of chromosome 22 has also been reported in 41% of GBM patients and is associated with tumoral progression [6], but no associations with survival rates were detected [85,89]. Furthermore, chromosome 4 alterations, as well as chromosome 12 alterations, have been described in the pathology of GBM [90].

#### 3.1.5. Neurofibromatosis Type 1 Gene (NF-1)

The tumor suppressor NF-1 encodes neurofibromin, which has been described as an inhibitor of the RAS/MAPK and PI3K-AKT-mTOR signaling pathways [91]. In up to 15% of glial tumors, NF-1 is inactive due to alterations such as mutations, deletion, or loss [50]. Low NF-1 expression and loss of function are strongly associated with the development of the mesenchymal GBM subtype [92]. Moreover, this gene could indirectly influence the aggressiveness of the mesenchymal subtype [93]. Although NF-1 correlation with GBM is still an open and debated research field, a recent study conducted by Chen et al. demonstrated that NF-1-inactivated GBMs exhibit a higher expression of neutrophil recruitment chemokines, leading to monocyte infiltration. Once the monocyte recruitment is stopped, GBM cells organize to allow neutrophils, which is why therapies that target individual chemokines fail. Thus, the authors suggested that combined therapeutics that simultaneously target neutrophils and monocytes could be very effective [94].

### 3.2. Molecular Biomarkers (Table 2)

#### 3.2.1. Amplification or Overexpression of Epidermal Growth Factor Receptor (EGFR)

Epidermal Growth Factor Receptor, also known as ErbB1/HER1, is the best-described member of the receptor tyrosine kinase (RTK) family, which comprises four receptors (ErbB1-4/HER1-4) [95]. This transmembrane receptor has a key role in major signaling pathways that are responsible for cellular survival, proliferation, migration, and the inhibition of apoptosis [75]. The signaling pathways involved in the aforementioned processes are PI3K/AKT, RAS/MAPK/ERK, the phospholipase C (PLC)/PKC, and the JAK/STAT pathway [96]. By hampering cellular apoptosis, they diminish TMZ’s effectiveness [97,98].

GBM frequently exhibits EGFR gene alteration or amplification, which results in high expression of mutations such as EGFRvIII or EGFR-wild-type (EGFRwt) [99]. EGFRvIII occurs after EGFRwt [100], and patients with overexpressed EGFRwt tend to have significantly lower survival rates compared to those without overexpression [99].

Overall, EGFR mutations are present in approximately 50% of all GBM patients. Of these mutations, roughly 40% are gene amplification [101]. The remaining samples comprise mutations, rearrangements, splicing site changes, and other modifications [101]. Approximately 40% of GBMs exhibit EGFR amplification, and all of these cases present an overexpression [75]. Gene amplification has been correlated with deletion mutations, especially EGFRvIII [75]. However, EGFRvIII is not a predictive element for survival. In cases with EGFR amplification, high expression of EGFRvIII is an important independent factor for poor survival [99]. Thus, it has been concluded that the most important indicator of poor prognosis is the overexpression of EGFRvIII in the presence of EGFR amplification, as it plays a major role in enhanced tumorigenicity [99].

The overexpression of EGFR is not only a key element involved in the proliferation, invasion, and survival of cancerous cells but also contributes to tumoral angiogenesis, and all of these characteristics diminish the chemotherapeutic and radiotherapeutic response in GBM [102]. Regarding the survival period, better outcomes were observed in EGFR-negative individuals after TMZ and radiotherapy [103].

EGFR amplification was observed for the first time in GBM as a result of a biomolecular examination [88]. EGFR amplification is represented by high levels of EGFR gene copies, while a lower number of copies is insufficient for diagnosis. Therefore, immunohistochemistry is not recommended, given the fact that it cannot adequately detect amplification [104]. EGFR amplification is not detected in other glial tumors with a better prognosis, as it has great specificity for very aggressive gliomas [104].

A better prognosis with longer survival periods was noted in the following instances: the presence of EGFRvIII in tumoral cells, the association of EGFRvIII/Ki67 ≤ 20%, the association of EGFRvIII/normal PTEN, and the association of EGFRvIII/methylated MGMT [105], while EGFRwt/TERT mutation predicts better outcomes than EGFR amplification [106]. However, poor prognosis (with a median survival of 12–16 months) was associated with the presence of EGFR amplification or TERT promoter mutations (TERTp-mut), and TERTp-mut is a powerful independent marker in GBM (unassociated with IDH), as is a low level of EGFR ligands [107]. Patients without either of these two alterations can have a survival interval ranging from 2 years (with IDH-wild-type) to 3 years (with IDH-mutation) [108].

Consistent with prior discussions, it has been stated that EGFR amplification along with mutation of TERT promoter and 7+/10− are alterations very often present in adult patients with IDH-wild-type GBM, and they can contribute to the upgrade of diffuse or anaplastic astrocytoma to wild-type GBM [88]. Thus, WHO CNS5 included this biomarker in the diagnosis criteria of GBM [11].

**Table 2:** Summary of described molecular biomarkers in GBM.

Biomarker Name	Identified Trait in GBM	Impact on GBM
**Mesenchymal Epithelial Transition Proto-Oncogene, Receptor Tyrosine** **Kinase (c-MET)**	Overexpression/spontaneous deregulation/induced or constitutive activation/elevated ligand production/mutations	Has an important contribution in tumoral cell growth, migration, and invasion. Overall survival rates were significantly shorter in patients with MET constitutive activation. Overexpression of MET was also correlated with a poor disease prognosis.
**Telomerase Reverse Transcriptase (TERT)**	Somatic mutations/alterations that could lead to overexpression. In GBM, the most common genomic alteration is represented by mutations of the promoter of TERT, but genomic rearrangements were also described, as well as transcript fusions or the amplification of the DNA.	Predicts survival outcomes and treatment response. Mutations of TERT promoter were correlated to a dismal prognosis. Point mutations of C228T and C250T are correlated with a worse prognosis and shorter survival rates. Alleles of rs2736100 and rs10069690 are correlated with a higher risk for developing GBM, while TERTp and rs2853669 predict shorter survival rates.
**Epidermal Growth Factor Receptor (EGFR)**	Genetic alteration/amplification.	It is an established diagnostic marker that can predict survival outcomes and treatment response. A therapeutic target that has been proven ineffective in GBM.
**Marker Of Proliferation Ki-67/Antigen Identified** **by Monoclonal Antibody** **Ki-67 (Ki-67)**	Overexpression. Differentially impacts cell proliferation.	A valuable proliferation marker, as lower Ki-67 index is associated with prolonged survival. Its overexpression can be a predictive marker for dismal prognosis and progression-free survival, regardless of cutoff value, region, and pathology type.
**Cellular Tumor Antigen P53/Tumor Suppressor Protein 53 (TP53)**	Mutation/homozygous deletion/the inactivation of the P53 tumor suppressor.	TP53 mutations are associated with a dismal prognosis and poor treatment response. Heightened activity of MGMT + low expression of TP53 was correlated to higher resistance to TMZ treatment. EGFR amplification + TP53 mutation coexistence carries a worse prognosis.
**B-Raf Proto-Oncogene, Serine/Threonine Kinase V600E (BRAF-V600E) mutation**	Mutation/hyperactivity that might cause cell arrest.	Predicts a relatively good prognosis, especially in pediatric population and young adults. Predicts an aggressive tumoral behavior in older patients with epithelioid subtype.
**Mismatch repair gene (MMR)**	Loss of MMR pathway/lack of expression/alterations of MMRpathway/MMR deficiency.	Resistance to temozolomide is driven by MMR defects. Alterations of MMR pathway/loss of MMR contribute to disease development and progression.
**Alpha-Thalassemia X-Linked Intellectual Disability (ATRX) Syndrome**	Mutation/loss.	A major predictor of disease progression. ATRX expression correlated to sodium–vitamin C cotransporter (SVCT2) is associated with a dismal prognosis in GBM. Loss of expression is correlated with longer survival rates.
**Neuron-glial antigen 2 (NG2/CSPG4)**	NG2 expression/overexpression.	NG2 expression identifies a highly aggressive GBM phenotype and is associated with resistance to chemotherapy and radiotherapy, while overexpression leads to neovascularization, vascular permeability, tumoral growth, and shorter survival rates.
**Hyaluronic acid receptor CD44**	Enhanced expression.	In GBM patients, enhanced CD44 expression predicts poor survival rates and is associated with different molecular subtypes. It is also associated with radioresistance.
**Oligodendrocyte lineage gene (OLIG)**	Overexpression/low expression.	OLIG2 is overexpressed in IDH1-mutant PDGFRα-amplified GBM and is correlated to a better prognosis. Low OLIG2 expression after adjuvant therapy will have a brief amount of time to recurrence and survival.
**Platelet-derived growth factor α-receptor (PDGFRα)**	Amplification.	NG2 and PDGFRα are key factors for cellular proliferation and motility. PDGF expression supports tumoral development of GBM, while amplification of PDGFRα significantly promotes tumoral aggressive behavior and is correlated to ATRX loss.

#### 3.2.2. Tumor Suppressor Protein P53 (TP53)

The TP53 gene encodes the tumor suppressor protein P53, which is an important regulatory factor in the cellular cycle, as well as a proapoptotic factor, and plays a major role in the DNA repair process [109,110]. One of the most frequently encountered molecular alterations in GBM is represented by the inactivation of the tumor suppressor protein P53 (in approximately 80% of cases) [111,112]. The deletion or mutation of the TP53 gene, as well as other errors regarding the P53 pathway, have been associated with susceptibility to gliomas [113] and sometimes co-occur with IDH mutations [111]. Thus, these reasonings indicate that P53 mutations play a key role in gliomagenesis, yet their role is to promote and develop the tumor rather than begin the formation process [112].

Since P53 can negatively regulate MGMT expression, its reactivation could have a major influence on tumoral growth and resistance to TMZ [114]. The intense activity of MGMT with low expression of TP53 was correlated with higher resistance to TMZ treatment [114]. Generally, TP53 mutations are associated with poor prognosis and poor treatment response [115].

Approaches aimed at therapeutically targeting TP53 have demonstrated limited results. Although restoring P53 produced tumoral cell apoptosis without affecting healthy cells and had a positive impact on the chemotherapeutical effect [68], some restoring strategies could in fact promote tumor formation [116]. Adenovirus-mediated therapy was also a field of interest, but further research is needed [117].

Given the fact that the main restriction of TP53 treatments was represented by the blood–brain barrier, several nanoparticles carrying this gene were developed and studied in the last decade, with promising results, as these nanoparticles targeted not only tumoral cells but also cancerous stem cells and had a positive impact on chemotherapy and survival rates [118,119].

#### 3.2.3. Telomerase Reverse Transcriptase (TERT)

The telomerase reverse transcriptase (TERT) gene is localized on locus 5p15.33 [120] and encodes a subunit of telomerase [121], an enzyme in charge of repairing the telomers to maintain their length [122]. By allowing cells to avoid senescence and keeping the telomere length, TERT maintains stability at the chromosomal level [120]. The majority of GBMs avoid cellular death through mutations of TERT promoter (TERTp) [123]. Healthy tissue has a low expression of TERT as well as low activity of telomerase, while cancerous tissue exhibits higher activity, thus acquiring immortality [124]. Therefore, it has been concluded that telomerase activity has a key role in GBM formation [123].

The most common genomic alteration in GBM is represented by mutations of TERT promoter, present in approximately 80% of IDH-wild-type GBM [125]. Genomic rearrangements were also described, as were transcript fusions, amplification of the DNA [126], and mutations of TERTp, which were correlated with a dismal prognosis [127].

The aforementioned genomic alterations are described as C228T in 75% of GBMs and C250T in 25% of cases and present worse prognoses and shorter survival rates [128]. Alleles of rs2736100 and rs10069690 were correlated with a higher risk of developing GBM, while cases with TERTp and rs2853669 had shorter survival rates [128]. Mutations of TERTp are important biomarkers for a poor prognosis, but this prognosis is affected by age and by the polymorphism of rs2853669 [129]. These mutations were described more frequently in IDH-wild-type GBM with EGFR amplification or gain of chromosome 7 or loss of chromosome 10 and were indeed correlated with a very aggressive evolution [12]. According to WHO CNS5, the diagnosis of GBM is also based on TERTp mutations [11], as they can be considered biomarkers for GBM behavior. The combination of these mutations with EGFR amplification and the gain of chromosome 7 or the loss of chromosome 10 gives more specificity [51].

Although TERTp mutations have great specificity for GBM, some other IDH-wild-type tumors can occasionally harbor these mutations. Thus, it is important to test for 1p/19q codeletion as well as for IDH mutations. Before considering TERTp mutation as a marker, IDH mutations must be ruled out [12,88].

This biomarker has been studied as a target for therapeutical purposes, but no effectiveness has been concluded yet [116,126].

#### 3.2.4. α, Thalassemia X-Linked Intellectual Disability (ATRX) Syndrome

In GBM patients, the mutation of alpha-thalassemia X-linked intellectual disability (ATRX) syndrome negatively impacts the restoration of DNA damage and is a major predictor of disease progression [130]. The inactivation of this biomarker in GBM is attributed to mutations, gene fusions, deletions, and combinations of these [131].

Alterations such as mutation or loss will lead to significant genetic instability and were reported in approximately 57% of recurrent GBMs. However, individuals with GBM who possess this mutation have shown better outcomes in comparison to patients with wild-type ATRX [130,132,133].

Given the fact that the mutation of ATRX and the codeletion of 1p/19q are mutually exclusive, it has been stated that ATRX mutation is more specific to astrocytoma IDH-mutant CNS WHO grade 4 [134]. However, Shang et al. identified a correlation between ATRX expression and sodium–vitamin C cotransporter (SVCT2) and concluded that this association leads to a dismal prognosis in GBM [130]. Furthermore, after analyzing the importance of ATRX mutation in patients with GBM, Gülten et al. concluded that the loss of ATRX expression in GBM was a biomarker for predicting better survival rates: median overall survival with loss of ATRX expression was 15 months, while median overall survival without loss of ATRX expression was 8 months [135].

Targeted therapeutic agents are investigated in patients with GBM and a loss of ATRX expression, with promising results for the future [136].

#### 3.2.5. Marker of Proliferation Kiel 67 (Ki-67)

When cells enter the mitotic phase, they express Ki-67 [137]. This protein is found in the cellular nucleus and is correlated with rRNA transcription, being exclusively ex-pressed by proliferating cells (normal and tumoral) [138]. The Ki-67 labeling index represents the percentage of cells that express Ki-67, and in GBM it is usually tied to the histological grade as a proliferative index [137].

The biomolecular roles of Ki-67 were distinct in different cellular stages. Hence, throughout the mitotic process, the protein covers the external chromosomal layer and is a key factor in the process of creating the perichromosomal surface. Subsequently, the protein shifts at the periphery of the nucleus, extending over the perinucleolar heterochromatin [139].

To assess the Ki-67 labeling index, immunohistochemistry techniques are being used. An index of less than 10% is considered negative, while an index of 10% or above is considered positive [140]. Although most studies considered a cutoff value of 10% as a significant predictor for survival rates, in GBM, a cutoff rate of 20% was associated with a notable effect on overall survival [141,142].

It has been revealed that the overexpression of Ki-67 is correlated with a dismal prognosis and progression-free survival regardless of its cutoff value, which concludes that the expression of Ki-67 can also be a predictive marker [143].

In patients with IDH-wild-type GBM, a higher expression of Ki-67 was reported and was associated with poor outcomes [144]. Armocida et al. concluded that Ki-67 > 20% in patients with IDH-wild-type GBM is a predictor factor for a dismal prognosis [145]. In a recent study about Ki-67 index use as a biomarker for survival in IDH-wild-type GBM patients treated with radiochemotherapy, Dumke et al. showed that a Ki-67 index ≤ 20% can be an independent factor that predicts and correlates with longer survival rates [144].

However, worth mentioning are some opposing results regarding the Ki-67 index and GBM. Unlike other authors, Wong et al. concluded that a Ki-67 index of equal or less than 22% can predict a poor prognosis (5-year survival of 5%), while a value greater than 22% is associated with longer survival rates (5-year survival of 30%) [143].

The quantity of tumoral cells from infiltrative edges in GBM could determine survival rates, as these edges carry a significant number of proliferative cells [146]. In patients with total tumor resection, Ki-67 is a key independent prognostic element, whereas in patients with subtotal resection, it is irrelevant [146]. Moreover, as the volume of the edema grows, the increase of Ki-67 is observed, and this could as well predict the prognosis [147].

Although all of these aforementioned studies concluded that Ki-67 can be an important biomarker for proliferation and survival in GBM, even in the preoperative settings [148], other studies did not find any correlation with prognosis [137,149,150], so its role as a prognostic factor is still debated.

#### 3.2.6. Mismatch Repair (MMR)

Mismatch repair is responsible for DNA repair, especially after replication errors. Thereby, it is responsible for maintaining the integrity of the genome [151]. While the loss of the MMR pathway will lead to microsatellite instability (MSI), in GBM, the importance of MMR is primarily defined by three aspects: resistance to alkylating chemotherapy is driven by MMR defects, MSI might predict immunotherapeutic response, and important proteins involved in MMR mediate TMZ sensitivity, especially in patients who lack MGMT expression [151]. Alterations of the MMR pathway are associated with increased tumor mutational burden (TMB) and response to immunotherapy [152]. High TMB is also correlated to a loss of MMR, and tumors that lack MMR do not have stability in telomeric DNA, which can contribute to disease development and progression [152]. This progression is also promoted by MMR deficiency that induces intratumoral heterogeneity [153].

It has been concluded that although mutations in MMR genes can influence TMB in GBM and IDH-mutated astrocytoma, the clinical outcomes are only relevant for the latter [152].

Several studies are approaching immunotherapeutic agents, but no clear contributions have been revealed [154,155].

#### 3.2.7. BRAF-V600E

The human BRAF gene (v-Raf murine sarcoma viral oncogene homolog B1) is involved in the formation process of B-Raf protein, responsible for signaling direct cell development [156], and has a key role in growth signal transduction [157]. The mutation is called V600E because of a shift from valine to glutamic acid at position 600 [156].

The BRAF serine/threonine protein kinase plays a vital part in the regulation process of the mitogen-activated protein kinase (MAPK)/extracellular signal-regulated kinase (ERK) signaling pathway [158]. Alterations of this proto-oncogene lead to unrestrained cellular multiplication and tumor formation [158].

In GBM, BRAF-V600E mutation has been associated with the epithelioid subtype GBM [159] in approximately 50% of cases [160] and is present in approximately 2% of all GBMs [161]. In older patients with BRAF mutation and epithelioid subtype, a worse prognosis has been reported [162].

Although this mutation has not proven any influence on chemotherapy or radiotherapy [158], the therapeutic activity of BRAF/MEK inhibitors has auspicious results [163], with near total GBM remission after 3 months of dabrafenib/trametinib treatment [163,164].

As a biomarker, BRAF-V600E can predict an age-dependable prognosis, as better survival rates were recorded in young adults [165].

#### 3.2.8. Mesenchymal Epithelial Transition Proto-Oncogene (MET)

Situated on locus 7q21-31, MET encodes the receptor tyrosine-kinase, and its receptor can be bound by its ligand, the scatter factor, or hepatocyte growth factor (HGF) [166]. In GBM, various processes will provide a route to atypical MET signaling. These processes are represented by mutations, constitutive activation, high auto or paracrine ligand production, or high expression, the last one being correlated with tumor grade [167].

High levels of MET were described in approximately 4% of GBMs, in which MET has a crucial role in the processes of invasiveness, tumor formation, and cellular renewal [168]. The induced or the constitutive activation of MET will start and support tumor cell growth while helping to evade apoptosis. Furthermore, MET cellular invasion and migration are mediated via focal adhesion kinase [167]. Overall survival rates were significantly shorter in patients with MET constitutive activation in comparison with patients who did not exhibit this modification [169]. The overexpression of MET was also correlated with a poor disease prognosis [170].

Due to its correlation to these tumoral processes, MET has been in focus not only as a biomarker for tumoral growth, migration, and invasion but also as a therapeutic target. However, in the adult population with GBM, the results were unsatisfactory, and further research is needed in order to assess its relevance [7].

#### 3.2.9. Neuron-Glial Antigen 2 or Chondroitin Sulfate Proteoglycan 4 (NG2/CSPG4), Hyaluronic Acid Receptor CD44, Olig-2, and Platelet-Derived Growth Factor α-Receptor (PDGFRα)

The last decades of research regarding molecular biomarkers have brought into the spotlight the fourth important glial cell type, called polydendrocytes, also known as neuron-glial antigen 2 or chondroitin sulfate proteoglycan 4 (NG2/CSPG4) [171]. It has been suggested that these cells can create neurons and oligodendrocytes and are taking part in the neural network [171]. In the adult population, of all the glial cells in the central nervous system, those expressing NG2 account for approximately 10% and are found dispersed in white matter and grey matter [172].

NG2-expressing cells in GBM are associated with significant cell proliferation, invasiveness, migration, and resistance to chemotherapeutic agents and radiotherapy, which raises interest in the area of targeted therapy, as this could slow or even hinder tumoral growth and improve therapeutic response [173,174]. NG2-expressing oligodendrocyte progenitor (OP) cells were recognized as the cell origin in adult GBM, and their high expression is correlated to highly aggressive behavior and shorter survival rates [174,175]. NG2 expression identifies a highly aggressive GBM phenotype, while overexpression leads to neovascularization, vascular permeability, tumoral growth, and shorter survival rates [176].

Hyaluronic acid receptor CD44 (HAR CD44) is also a chondroitin sulfate proteo-glycan, like NG-2, and is a major factor involved in tumoral progression and metastasis. HAR CD44 has been shown to be a marker for cancer stem cells (CSC) in GBM [177]. Although experiments correlated high expression of CSC markers to radiosensitivity, CD44 was the only exception, and it was associated with radioresistance [178].

Hyaluronic acid can be found abundantly in the stroma of GBM tumoral tissue, and it is involved in cell proliferation and invasion. In GBM patients, enhanced CD44 expression predicts poor survival rates [178,179] and is associated with different molecular subtypes [180]. It has been suggested that targeted therapy focused on CD44 inhibition could bring promising results [181]. However, further research is needed in order to draw conclusions.

Other than NG2 proteoglycan, the vast majority of gliomas express other markers typical for OP, such as Olig-2 and PDGFRα, while NG2 and PDGFRα are key factors for cellular proliferation and motility [182]. PDGF expression supports the tumoral development of GBM, while the amplification of PDGFRα significantly promotes tumor aggressive behavior and is correlated to ATRX loss, leading to further research regarding therapeutic target opportunities in this field [54,131].

Oligodendrocyte lineage gene (OLIG) expression has been demonstrated in GBM, and it was of great help for a better understanding of this disease [183]. A thorough analysis of GBM samples concluded a great number of OLIG-2-positive cells [184]. Along with PDGFRα and NKX2-2, the overexpression of OLIG-2 was mostly detected in the proneural subtype. Furthermore, it has been demonstrated that the amplification of OLIG-2 induces tumor formation in GBM and promotes cellular proliferation [112]. Among all of the glioma stem cells, the only ones related to the regulation of the cell cycle are OLIG-2 and cyclin D2 [183].

The expression of OLIG-2 is frequently nuclear and very rarely cytoplasmic, and it significantly decreases in patients with GBM recurrences [183]. Recent studies concluded that patients with low OLIG2 expression after adjuvant therapy will have a shortened recurrence time and survival rate [183].

### 3.3. Circulating Biomarkers (Table 3)

Despite significant research focusing on circulating biomarkers in GBM over the past 10 years, minimal progress has been made in the field [185].

The concept of liquid biopsy has been proposed as a way to assess real-time GBM dynamics and response to therapy, especially as a solution to the limitations of neuroimaging modalities and tissue biopsy [186].

Cerebrospinal fluid (CSF), blood, and urine are used as biological fluids to identify and investigate GBM biomarkers. These biomarkers have their origins in the tumor sample, and their analysis is called liquid biopsy [187]. The liquid biopsy not only provides information about therapeutic response and real-time dynamics but can also provide important details about tumor features and recurrence [188]. The biological markers detected in liquid biopsies are mainly represented by circulating tumor cells (CTC), circulating tumor nucleic acids, extracellular vesicles (EV), and circulating proteins [187,188,189].

**Table 3:** Summary of described circulating biomarkers in GBM.

Biomarker Name	Identified Trait in GBM	Impact on GBM
**Circulating tumor cells (CTC)**	GBM cells circulating into the bloodstream.	Could be used as biomarkers for response to radiotherapy and also to assess for recurrence. Can differentiate recurrence from radionecrosis. They are more frequent in GBM patients with extracranial metastases.
**Circulating cell-free DNA (ccfDNA)**	DNA particles released by GBM cells.	Could be used to distinguish tumor recurrence from radiation necrosis. Can be used as a biomarker for the tumoral activity or tumoral burden. Elevated levels of cfDNA could be correlated to worse outcomes and shorter survival rates and can be a biomarker for treatment response. Increased plasma cfDNA concentrations in a subcategory of GBM patients is correlated independently with a dismal prognosis.
**Cell-free RNA (cfRNA)**	Elements passively originating from cells that are necrotic or apoptotic or actively originating from EV pathways.	Circulating miRNA could be biomarkers for disease assay and miR-21, 128, and 324 could predict treatment response to chemotherapy.
**Extracellular vesicles (EV)**	Nanosized membrane surrounded particles of various shapes that are released by GBMs.	Diagnostic and prognostic biomarkers and useful in the targeted therapeutic strategy.
**Circulating Proteins**	Tumor cell–derived proteins have been found in peripheral blood or CSF samples in patients diagnosed with GBM.	GFAP is correlated with tumor volume and histopathological features. YKL-40 is associated with poor survival rates and is an important prognostic biomarker that could be used as a target for anti-glioma therapy. LRG1, CRP, and C9 were correlated with tumor size.

#### 3.3.1. Circulating Tumor Cells (CTCs)

While their phenotypes shift after epithelial-to-mesenchymal conversion, CTCs are cancerous cells originating in the primary tumor and circulating into the bloodstream [190]. CTCs can be found in CSF, urine, and blood and are significantly involved in the process of tumor dissemination [191].

Notwithstanding, GBM invasiveness is usually limited to the brain. It can very seldom metastasize through CTC with mesenchymal features. Furthermore, the metastases are mainly mesenchymal and exhibit supplementary mutations that were not detected in the primary lesion [192].

In GBM, the amount of CTC in postradiotherapy patients was significantly lower in comparison to preradiotherapy patients, which means that they could be used as biomarkers for response to radiotherapy and also to assess for recurrence [193]. Likewise, CTCs were successfully used to assess treatment response, with slightly superior outcomes to MRI monitoring, and significantly contributed to differentiating the recurrence of radionecrosis [194].

Circulating glial fibrillary acidic protein (GFAP)-positive putative cells were detected in the peripheral blood of patients with GBM before and after tumoral excision. Approximately 6% of cases were detected before the surgical excision but not after excision, and in 7.5% of cases, GFAP-positive CTCs were only detected after tumor excision, but not before it. This concluded that CTCs are more frequent in GBM patients with extracranial metastases [195].

#### 3.3.2. Circulating Cell-Free DNA (ccfDNA)

DNA particles released by cancerous cells are known as ccfDNA, but these fragments are also present in healthy individuals, and they originate from inflammatory activity or apoptosis [196]. The ccfDNA is the most attainable source of tumor cells, specifically circulating tumor DNA (ctDNA), which is a more shattered version in comparison to ccfDNA [197]. The amount of ctDNA in GBM patients can be used as a biomarker for tumor activity or tumor burden, as its levels were lower at one-month-postsurgical excision [197].

Although it has been demonstrated that ctDNA is elevated in most primary brain tumors, GBM was an exception, with decreased levels of serum ctDNA [198]. However, recent research discovered that elevated levels of cfDNA from preoperative to postchemotherapy and postradiotherapy could be correlated to worse outcomes and shorter survival rates, concluding that cfDNA might be a biomarker for treatment response [199]. It is also worth mentioning that in GMB patients, the main part of plasma cfDNA does not originate in the actual tumor, and additional studies are needed to establish the tissue of origin [199]. Nevertheless, plasma cfDNA concentrations are increased in a subcategory of patients, and this concentration is correlated independently with a dismal prognosis [199].

While many studies approached the subject of ccfDNA in GBM, its actual role has not yet been fully understood, and further research is needed to plainly understand it [200].

#### 3.3.3. Cell-Free RNA (cfRNA)

Cell-free RNA represents a molecular category passively originating from cells that are necrotic or apoptotic, or actively originating from EV pathways. They are generally classified into coding and noncoding RNA [6]. Noncoding RNA is divided into short and long noncoding RNA, and the most researched long noncoding RNA is represented by microRNA (miRNA). While in GBM, circulating miRNA could be a biomarker for disease assay, miR-21, 128, and 324 could predict treatment response to chemotherapy [201]. It is worth mentioning that these miRNA subcategories can regulate the MET pathway, while the latter can regulate the expression of miRNA sub-categories in order to offer self-feedback and dysregulation at any level that would lead to the evolution of cancer [167]. Given the fact that the miR-134 subtype can decrease cellular proliferation and invasion and hinder GBM development, the suppression of miR-134 has been suggested as a new instrument that explains how receptor tyrosine kinases support cellular signaling output in this disease [167].

Although it has been stated that cfRNA has increased stability, this can be attributed to its inclusion in extracellular vesicles and to its connection to other proteins [202].

Despite the fact that a large number of studies approached the subject of cfRNA in GBM, almost all of them are exploratory, thus raising the need for further research.

#### 3.3.4. Extracellular Vesicles (EVs)

Extracellular vesicles represent a heterogeneous category of nanosized membrane-surrounded particles of various shapes that are released by GBMs [203]. They have the capacity to communicate between tumor compartments and the tumor microenvironment. They can be detected and obtained from CSF and from blood. Their most important features are that they contain large amounts of tumor-derived molecules and they can protect themselves from nucleases and proteases [203].

The detection of EVs in CSF and blood is also due to their capacity to cross the blood–brain barrier in normal and pathological conditions [204].

Recent studies showed that EVs can transfer multiple microRNA components, as well as EGFRvIII mRNA/protein, or PTEN, and increase malignancy in GBM cells [205]. Moreover, high levels of microRNA were associated with GBM progression, while the overexpression of miR-21 was correlated to invasiveness. It is worth mentioning that EV messenger RNA is associated with neovascularization, histone transformation, immunological responses, cellular proliferation, and migration [206,207].

It has been stated that in GBM, EVs significantly contribute to cellular proliferation and invasion, as well as aggressiveness and resistance to treatment, and that tumor cells produce greater amounts of EV, which communicate with neighboring cells and promote immunosuppression and proliferation [208,209].

In conclusion, EVs are small membrane-bound vesicles secreted by cells into the extracellular environment and which play an important role in intercellular communication by transporting various molecules [210]. Recent studies have shown that GBM cells release exosomes that can promote tumor invasion and migration, facilitate the malignant transformation of normal cells, increase vascular supply, and induce therapy resistance. Exosomes are valuable not only as diagnostic and prognostic biomarkers but also for targeted therapeutic strategies [206,211].

#### 3.3.5. Circulating Proteins

Pathological processes in GBM can extend beyond the brain by involving the systemic circulation of proteins. Even though there are no glioma-specific proteins, aberrant circulating levels of some tumor cell–derived proteins have been found in peripheral blood or CSF samples in patients diagnosed with GBM [6,212], with some of them possibly being relevant in the context of this disease.

Glial fibrillary acidic protein (GFAP) serum levels are frequently detected in GBM patients. The plasma concentration levels of this protein are correlated with tumor volume and histopathological features, helping to differentiate between GBM and other brain tumors [213,214].

YKL-40 serum marker is highly expressed in GBM patients and is associated with poor survival rates. It is considered an important prognostic biomarker that could be used as a target for anti-glioma therapy [215,216].

Another study looking at blood biomarkers with an impact on GBM biology found that leucine-rich alpha-2-glycoprotein (LRG1), C-reactive protein (CRP), and complement component C9 (C9) were correlated with tumor size [217].

Exosomes are also cited as having a major contribution to GBM diagnosis and prognosis. However, this subject has already been approached above (extracellular vesicles).

In conclusion, circulating proteins in GBM can provide information about the tumor microenvironment and disease progression, as well as potential therapeutic targets.

### 3.4. Circulatory Biomarkers (Table 4)

Given the fact that GBM is a highly vascularized tumor and exhibits atypically elevated levels of vascular endothelial growth factor (VEGF), therapies targeting this element have been studied [218]. Circulatory biomarkers have been proposed for prognosis and for anti-angiogenic therapies [218].

Vascular endothelial growth factors (VEGFs) are categorized as polypeptides and are members of the VEGF/PDGF (platelet-derived growth factor) class of cystine-knot signaling molecules [219]. VEGF (VEGF-A or vascular permeability factor) was described for the first time by Sanger et al. [220] and is known as the most representative archetypical member of its category, the main indispensable regulator of angiogenesis (including embryonic angiogenesis), and an essential element involved in the process of neovascularization in cancer [219]. In GBMs, the identified trait of VEGF is represented by overexpression [221]. The genetic processing of its receptor revealed significant developmental hindrances of GBM, and the need for better alternatives led to pharmacologic anti-VEGF therapy such as bevacizumab [222]. However, while bevacizumab showed as a main benefit a reduction in steroid requirements, there was no improvement regarding overall survival rates [222,223]. Other anti-angiogenic agents, such as sunitinib, sorafenib, and enzastaurin, demonstrated modest and limited results [224,225,226]. Although these treatments did not influence survival rates in patients with GBM, their positive effect on progression-free survival indicates a certain benefit [227].

Plasma levels of the VEGF-121 isoform could be a promising biomarker in GBM, and they are associated with treatment response and toxicity following treatment with bevacizumab [228]. Higher plasmatic levels of the VEGF-121 isoform were associated with worse progression-free survival and overall survival [229,230]. VEGF121 binds to plasma-soluble VEGFR-1 (PsVEGFR-1), and experimental studies concluded that their interconnection is dependent on multiple microenvironmental factors [231]. High expression levels of VEGF-A and of the VEGFR-1 ligand PIGF are inversely correlated with survival [232]. It has been stated that PIGF is involved in the neovascularization process, but it is not an essential element [233]. Moreover, PsVEGFR-1 is correlated with treatment response and toxicity after the administration of bevacizumab [234]. The genetic polymorphism of VEGFR-2 might prognosticate response to antiangiogenic therapy in patients with GBMs [235].

Loureiro et al. concluded that in most cases of GBM, there is an overexpression of VEGF-C and VEGF-D and its receptors, but there is no association between these findings and survival rates [218]. However, the overexpression of these polypeptides, as well as of VEGFR-2 and 3, could be potential targets for further research [218].

Another proposed biomarker for antiangiogenic activity is represented by circulating endothelial cells (CECs), which are evolved cells resulting from vascular injury and are a distinct entity from circulating tumor cells [236]. The number of CECs represents a marker for endothelial destruction [237]. However, the association with survival rates is still controversial [238], but their detection has a prognostic role at diagnosis [239].

Despite ongoing debate about the prognostic usefulness of programmed death-ligand 1 (PD-L1), recent studies have shown a correlation between its expression and survival rates in GBM [240,241]. PD-L1 expression was demonstrated in approximately 88.0% of GBMs [242], and high expression was correlated to a dismal prognosis [240,241], as well as with CD3-positive T-cell invasion and IDH-1-wild-type status [241]. The probable efficacy of anti-PD1/PD-L1 agents as monotherapy, neoadjuvant therapy, or even combinations, was suggested [243]. Currently, the PD1/PD-L1 pair is involved in GBM biological stratification, while immunological therapy aims to restore the work of former T-cells [244].

**Table 4:** Summary of described circulatory biomarkers in GBM.

Biomarker Name	Identified Trait in GBM	Impact on GBM
**Vascular Endothelial Growth Factor (VEGF)**	Overexpression	Biomarker for response to anti-angiogenic therapy, especially in recurrent GBM.
**Soluble Vascular Endothelial Growth Factor Receptor 1 (sVEGFR1) and Soluble Vascular Endothelial Growth Factor Receptor 2 (sVEGFR2)**	Overexpression	Higher levels are correlated with lower survival rates in patients receiving anti-angiogenic therapy and longer progression-free survival after treatment with tandutinib. A potential biomarker for resistance to anti-VEGF treatment. The sVEGFR2 can predict bevacizumab benefits. Genetic polymorphism of VEGFR-2 might prognosticate response to antiangiogenic therapy.
**Placental Growth Factor (PIGF)**	Involved in neovascularization.	Correlated to therapeutical response and survival period. Associated with longer progression-free survival in patients treated with cilengitide and cediranib in combination, and tandutinib.
**Circulatory Endothelial Cells (CEC)**	Evolved cells resulting from vascular injury.	Proposed biomarker for antiangiogenic activity.
**Programmed Cell Death Protein 1 (PD-1) and Programmed Cell Death Ligand 1 (PD-L1)**	Upregulated/high expression.	Associated with poor survival rates. Could be a prognostic predictor of treatment response to GBM vaccines and efficacious therapeutic target.

### 3.5. Other Biomarkers (Table 5)

#### 3.5.1. Extracellular Signal-Related Kinases 1 and 2 Phosphorylation (p-ERK)

ERK1/2, the final elements in the MAPK cascade, have recently been in the spotlight due to their association with cancer development through signaling dysregulations [245]. It has been stated that ERK1 and 2 possess significant similarities, thus being referred to collectively [245]. While they can activate the mammalian target of rapamycin complex 1 (mTORC1) and Hypoxia-Inducible Factor Alpha (HIFα), they are also important factors in the process of cellular development, differentiation, proliferation, and migration [245].

It has been shown that the activation of the MAPK/ERK signaling pathway can be associated with response to PD-1 inhibitors in patients with recurrent GBM and that p-ERK, which is considered a marker of this pathway activation, can be a biomarker that predicts survival in these patients [246]. Although just a small group of patients will benefit from PD-1 blockade, these positive results are encouraging, given the prolongation of survival rates in patients with recurrent GBM [246].

Intravenous nivolumab combined with neurosurgical resection and intracerebral ipilimumab monotherapy or ipilimumab+nivolumab showed promising results in patients with IDH-wild-type GBM, as increased p-ERK was correlated with prolonged survival rates [247]. Thus, the role of p-ERK as a biomarker for predicting survival rates in patients with GBM treated with immune checkpoint inhibitors has been proposed lately [247].

Another group of patients that may benefit from immune checkpoint inhibitors has been described by Hadad et al.: patients with a newly described GBM subtype [248]. The authors discovered a new subtype of GBM in 2% of patients after analyzing the genome of 459 individuals with treatment-naïve IDH-wild-type glioblastomas. These patients exhibited somatic hypermutation or ultrahypermutation and DNA replication repair deficiency. They had a median age of 50 years, and all had giant-cell variants of GBM. Those who were treated with immune checkpoint inhibitors had a survival rate greater than 3 years (median survival of 36.8 months in comparison to 15.5 months in the rest of the patients) [248]. Although patients in this newly detected subgroup did not exhibit EGFR amplification, +7/−10, and TERTp or CDKN2A mutations, they had mutations in TP53, NF1, PTEN, ATRX, SETD2, and PDGFRα. The authors concluded that these patients can be included in the new GBM subcategory named “De novo replication repair deficient glioblastoma, IDH-wildtype” [248].

**Table 5:** Summary of other described biomarkers in GBM.

Biomarker Name	Identified Trait in GBM	Impact on GBM
**Extracellular signal-related kinases 1 and 2 phosphorylation (p-ERK)**	Signaling dysregulations.	Important factors in the process of cellular development, differentiation, proliferation, and migration. Can be a biomarker that predicts survival.
**Herpes simplex virus 1 (HSV1) serology**	Oncolytic herpes virus 1 can alter the GBM TME.	Positive HSV1 serology was highly predictive of treatment response, as patients who were positive before administration of the oncolytic virus had higher median survival rates when compared to previous reports.
**Delayed-type hypersensitivity reactions (DTH)**	In patients with GBM, an intradermic reaction to a specific antigen (oncolytic anti-EGFRvIII peptide vaccine) can be identified.	Minimally invasive biomarker that could be used as a predictor of immune response in clinical trials.
**Hypoxia**	Improves proliferation/prevents the degradation of IDH-mutated forms.	Hypoxia is correlated to aggressiveness, resistance to treatment, and recurrent behavior.
**Cytokine levels**	Proinflammatory or immunosuppressive role that will help them escape the immune system and contribute to tumoral growth and aggressiveness.	Potential biomarker for treatment response to GBM vaccines and a promising therapeutic target.

#### 3.5.2. Herpes Simplex Virus 1 (HSV1) Serology

A viable way of altering the tumoral microenvironment in GBMs is represented by oncolytic viruses. Currently, there is only one oncolytic virus approved as immuno-therapy in patients with GBM, in Japan, known as oncolytic herpes virus G47, with promising results, as a complete response was recorded in one patient at the 2-year MRI follow-up [249].

Another recent phase I clinical trial reports its results for patients with recurrent GBM who were treated with CAN-3110, which is an oncolytic virus derived from HSV1 [250]. Unlike any other previous reports, this study observed that CAN-3110 was detected even after months and years in almost half of the injected individuals, which may suggest its ongoing replication, as well as the persistence given by the expression of ICP34.5 with antitumoral activity [250]. The positive HSV1 serology was highly predictive of treatment response, as patients who were positive before administration of the oncolytic virus had higher median survival rates when compared to previous reports (14.2 months versus 6–9 months) [250].

#### 3.5.3. Delayed-Type Hypersensitivity (DTH) Reactions and Genomic Biomarkers

Delayed-type hypersensitivity reactions represent an intradermic reaction to a specific antigen. They have been suggested as a potential biomarker in GBM patients treated with the oncolytic anti-EGFRvIII peptide vaccine rindopepimut, as the intra-cutaneous vaccination can develop a specific humoral response that can be identified in the CSF and in DTH [251,252]. Among patients who exhibit DTH, survival rates were longer [251]. While DTH does not directly correlate with overall survival rates in GBM patients, it can serve as an unspecific marker to assess T-cell activity, as the reaction increases over the course of the vaccination [253]. However, no recent studies regarding this biomarker have been published, so currently, these findings remain a matter of the past.

Patients with newly diagnosed GBM may benefit from clinical and genomic predictors of adverse reactions. In a recent study, Lim-Fat et al. concluded that patients with GBM may have many potential complications (e.g., seizures, venous thromboembolism) and tried to correlate these with the type of patients by analyzing their genomic alterations [254]. Although the study did not conclude many major predictors for adverse reactions in patients with GBM, it did help identify a predictor, particularly for seizures. Individuals with alterations in EGFR, SMARCA4, GNA11, and BRD4, and with TCF3 gain of function and SETD2 loss of function, had a higher risk of seizures [254]. The authors mentioned that a great limitation of the study was represented by including only IDH-wild-type GBM patients, given the fact that patients with IDH alterations were more prone to seizures [254].

#### 3.5.4. Reactive Oxygen Species (ROS), Hypoxia, and Autophagy

The physiological equilibrium between reduced equivalents and reactive oxygen species is known as cellular redox status [255], while the imbalance between the formation of ROS and the capacity to eliminate them is known as oxidative stress [256]. Although ROS provide cellular protection, when available in excess amounts, they will contribute to the generation of other highly reactive species [256]. In humans, the aforementioned imbalance leads to cellular and molecular damage, contributing to the development of various diseases [255,256]. The major site of ROS production is represented by mitochondria, and the consequence of high mitochondrial levels of ROS and Calcium 2+ is membrane damage, which leads to even more ROS [255]. In GBM, the process that sustains malignancy is the defective mitochondrial coupling of ROS [257].

HIF1 is a key factor that orchestrates the adaptation of cells to a low amount of oxygen [258]. Although this dimeric protein is significantly involved in the response to the hypoxic state, homeostasis, anaerobic metabolism, immunological responses, and angiogenesis, it can also play a major role in cellular proliferation and survival in numerous cancerous diseases [259]. Given its important role in cancer, therapeutical agents targeting HIF1 inhibition could have a major impact on preventing the proliferation of cancerous cells and, therefore, cancer progression [259]. In GBM, hypoxia is a crucial element involved in the tumoral microenvironment, and HIF1α can trigger its occurrence [6].

It has been stated that the hypoxic state will activate the expression of biomarkers such as VEGF and sVEGFR-1 [260], and extensive hypoxic areas may frequently supply perivascular dwelling for self-restoring glioma-initiating cells (GICs), which can lead to more aggressive tumors with recurrent behavior and resistance to treatment [261,262,263].

In human GBM, positive HIF1α expression was correlated to a dismal prognosis, the overexpression was associated with unsatisfactory therapeutic response and resistance to radiotherapy and chemotherapy, while the negative expression predicted better outcomes [264].

As a result of the hypoxic state, a cytoprotective process called autophagy has been described [33]. This mechanism is actively involved in maintaining homeostasis and the integrity of the genome, and in GBM, it is activated in GICs as a result of cytotoxic treatments [33]. Hence, autophagy is described both as a defense mechanism for cells and an oncogenic process, but the precise shift between these two states is still debated. In GBM, autophagy mediates treatment resistance to anti-angiogenic chemotherapies, but its role in radiotherapy is dual, providing protection or inducing cytotoxic effects [265,266].

Autophagy could ease the degradation of zinc finger proteins SNAI1 and SNAI2 and therefore suppress the cellular migrative and invasive features of GBM [267]. As a potential therapeutic target (inhibition or induced autophagy), it has shown promising results that need further investigation [268].

#### 3.5.5. Cytokine Levels

Cytokines are small molecules with various functions that can contribute to tumor growth and aggressiveness due to their pro- or anti-inflammatory function or to their immunosuppressive role, which helps them escape the immune system [269]. Hence, determining the levels of cytokines may provide important information about the intricate connection between therapy and proinflammatory and immunosuppressive roles in GBM, giving them the potential value of a biomarker [43].

A significant immunological reaction was reported after the autologous dendritic cell vaccine by evaluating serum cytokine levels, translated by an increased production of interferon-gamma (IFN-γ). Nevertheless, no association was found between cytokine proliferation and survival rates [270,271].

The role of cytokines as immunotherapeutic targets may offer an avenue for promising results, especially when it comes to transforming growth factor beta (TGFβ) [272] and colony stimulating factor 1 (CSF1) [273], but this field needs further research.

### 3.6. New or Potential Biomarkers for GBM (Table 6)

#### 3.6.1. Aurora Kinase A

It has been stated that aurora kinase A (AURKA) has a major role in cell cycle progression, tumor development, and treatment resistance, and it is associated with a dismal prognosis. Its most important pathological trait is represented by overexpression/upregulation, and nowadays, multiple inhibitors are being evaluated for different cancers [274]. Alisertib, which is an inhibitor of this serine/threonine kinase, demonstrated significant antiproliferative activity against GBM, especially when combined with TPI 287 and GBM stem cells, interfering with mitotic slippage in GB30 neurosphere cells [275]. It seems that alisertib can activate EGFR signaling in GBM, which subsequently increases LPCAT1 expression and alters the phospholipid composition of GBM [274]. Although, in other types of cancer, LPCAT1 was found to sustain malignant cells, in GBM, the increased expression was found to inhibit cellular proliferation. Other than alisertib, AMG900 and AT9238 (a small-molecule inhibitor) also demonstrated benefits in GBM, but further research is needed [274].

The inhibition of AURKA in GBM could also be beneficial when associated with high radiation doses, suppressing tumoral growth [276], and the miR-124–AURKA axis can regulate tumoral growth and chemosensitivity. Thus, by inhibiting AURKA, miR-124 will not only block GBM development but even potentiate chemosensitivity [277]. Another promising, highly synergic combination for GBM treatment is represented by dual AURKA/BET inhibition, respectively alisertib and birabresib, a pan-BET inhibitor [278]. In addition, the dual inhibition of aurora kinases and Lim kinases by F114 demonstrated promising results in a recent study regarding GBM [279].

It is worth mentioning that in GBM, POLE2 can regulate AURKA-mediated FOXM1 ubiquitination, which may be the basis of molecular treatment in this disease [280].

All these results show that the acute and chronic inhibition of AURKA, as well as dual inhibitions, could be beneficial in patients with GBM and that this biomarker could be a significant therapeutic target in the future [281].

**Table 6:** Summary of described new or potential biomarkers in GBM.

Biomarker Name	Identified Trait in GBM	Impact on GBM
**Aurora kinase A (AURKA)**	Overexpression/upregulation.	Inhibition of this biomarker demonstrated significant antiproliferative activity against GBM, suppressing tumoral growth and sustaining chemosensitivity.
**Nuclear division cycle 80 (NDC80), Kinesin superfamily protein 4A (KIF4A), and nucleolar and spindle-associated protein 1 (NUSAP1)**	Overexpression.	They have a crucial role in tumorigenesis. Their inhibition induced the arresting and apoptosis of GBM cells. They can be precise diagnosis and prognosis biomarkers.
**Dickkopf WNT signaling pathway inhibitor 3 (DKK3)**	Downregulation/expression.	DKK3 expression can significantly impact immunosuppression and predict a dismal prognosis. Indirectly involved in tumoral proliferation.
**Thrombospondin-1 (TSP-1)**	Overexpression	Can predict survival rates and is correlated with tumoral expansion and invasiveness, as well as treatment resistance.
**Podoplanin (PDPN)**	Overexpression	High amounts of PDPN are correlated with shorter survival rates and aggressiveness. Could be a promising therapeutic target, blocking GBM development and progression.
**Annexin A1 (ANXA1) and collagen type VI alpha 1 chain (COL6A1)**	Upregulated in high-risk GBM patients.	Involved in neovascularization and associated with a worse prognosis.

#### 3.6.2. Nuclear Division Cycle 80, Kinesin Superfamily Protein 4A, and Nucleolar and Spindle-Associated Protein 1

In addition to AURKA, another three hub genes were discovered to be important in GBM: nuclear division cycle 80 (NDC80), kinesin superfamily protein 4A (KIF4A), and nucleolar and spindle-associated protein 1 (NUSAP1). These genes were significantly overexpressed in comparison to healthy individuals and play a crucial role in tumor formation. Patients with GBM and low expression of these genes demonstrated better prognoses, with longer survival rates. Methylation levels were lower in GBM patients, while hypomethylation was the cause of overexpression and tumorigenesis. Regarding these genes, JNJ-7706621 inhibitor demonstrated significant benefits by inducing the arrest and apoptosis of GBM cells. Recent studies demonstrated that these genes could be precise diagnosis and prognosis biomarkers, as well as potential therapeutic targets, in GBM patients [282,283].

#### 3.6.3. Dickkopf WNT Signaling Pathway Inhibitor 3 (DKK3)

In GBM, the WNT pathway is aberrantly activated, promoting tumoral development. This pathway has a vital role in different cellular processes such as proliferation and differentiation and is also involved in tumoral invasiveness. This specific pathway can be regulated by the dickkopf family of proteins, particularly DKK3, which has been proven to be its most significant modulator [284], acting as a crucial antagonist of the WNT/BETA-catenin pathway [285].

Casili et al. demonstrated that DKK3 is present in a small amount in GBM patients, concluding the downregulation of this biomarker in tumoral growth. The study also shows that the antiproliferative feature of this biomarker is due to the modulation of caspase-9-dependent apoptosis. Furthermore, the overexpression of DKK3 can induce apoptosis through another pathway, termed c-Jun-NH2-kinase (JNK), which is involved in the tumoral growth of GBM. Additionally, the TLR-4 gene, which is present in the GBM TME and is involved in numerous tumoral processes, could be an important therapeutic target related to DKK3 [286]. TLR-4 deficiency preserves not only DKK3 but also the expression of claudin-5, sustaining apoptosis, and thus slowing the growth of GBM cells. The TLR-4 pathway is correlated to the expression of the WNT pathway, and the downregulation of the first one would modulate the WNT-DKK3–claudin-5 axis and damper GBM proliferation [286].

Recent findings suggest that in GBM patients, DKK3 is more likely to be correlated with the expression of various WNT/β-catenin pathway-related genes and is associated with immunological downregulation, while DKK3 expression can significantly impact immunosuppression and predict a dismal prognosis [287,288].

In conclusion, DKK3 could be a significant therapeutic target for patients with GBM, yet further research is needed regarding the matter.

#### 3.6.4. Thrombospondin-1

Thrombospondin-1 (TSP-1) is an element found in the ECM and has an important role in cancer pathology. In GBM, the expression of TSP-1 is very high and is correlated to survival rates and cellular invasion and expansion, while having the greatest connectivity when it comes to the invasive compartment [289]. The association with tumoral expansion and invasion is also proved by TSP-1 inhibition after a decrease in mass volume. However, the inhibition of this biomarker would not only be beneficial when it comes to invasiveness but also when it comes to the vascular compartment [289].

Another recent study identified this biomarker as a pro-invasive gene localized within the invadopodia that cover GBM cells. The study concluded that its overexpression is correlated to cellular migration and MMP-2 secretion, while its inhibition will damper these processes [290].

It has been demonstrated that TSP-1 expression is also associated with the expression of other immune-related genes, such as MHC I and II or STAT1, as well as with immune cell infiltration such as Tregs, a subcategory of T cells. Thus, the overexpression of this biomarker leads to an increased immunological response, which might predict a crucial feature in immunosuppression [291,292].

GBM cells can form extensions that connect tumoral cells with each other or even with other cells from the TME. These extensions are called microtubes [293]. Joseph et al. found that the TGF-β pathway is overly activated in GBM, and its inhibition is associated with less tumoral invasion and microtube formation. The study concluded that by blocking the pathway and its downstream mediator, which is TSP-1, the microtube-driven invasion and treatment resistance complex will break [294].

Although some authors agree on the role of TSP-1 in GBM, the final approach might remain controversial. Further studies are needed in order to discover the real potential of this biomarker [295].

#### 3.6.5. Podoplanin, Annexin A1, and Collagen Type VI Alpha 1 Chain

In the last decade, a special receptor has been discovered, termed podoplanin (PDPN). This transmembrane glycoprotein expression is influenced by other tumor promoters such as TPA, RAS, and Src, and can be detected in various human cancers. The main feature of PDPN is to help cancer progression by blocking the death of tumoral cells and by sustaining cellular migration. High amounts of PDPN are correlated with aggressiveness and shorter survival rates [296]. As a biomarker of cancers, PDPN is correlated with more aggressive behavior, tumoral thickness, and invasiveness [297].

Notwithstanding these findings, new research regarding this biomarker is still ongoing. In addition, a new CTC chip made out of resin and coated with PDPN antibodies is being studied in order to find and capture metastases [296].

PDPN has potential not only as a biomarker in GBM but as a therapeutic target as well. When it comes to its extracellular domain, CAR-T cells, antibodies, and lectins demonstrated promising results by blocking GBM development and progression [298,299]. Furthermore, antibodies have been proven effective even in disrupting PDPN–CLEC-2 interactions, as this pair sustains tumoral cell migration and extravasation [300]. Regarding the intracellular domain, protein kinase A and cyclin-dependent kinase 5 demonstrated the ability to inhibit tumoral cell migration as well [301].

In addition to PDPN, another two genes have been described: annexin A1 (ANXA1) and collagen type VI alpha 1 chain (COL6A1). These three genes interact with each other, and it is possible that they are involved in GBM neovascularization. Moreover, a recent study by Wan et al. demonstrated that these three genes are notably upregulated in high-risk GBMs, are correlated with a worse prognosis, and, as biomarkers in GBM, they could predict prognoses and be therapeutic targets [302].

## 4. Conclusions

Despite significant progress in the therapeutical field of GBM, patients still have poor prognoses, with short survival rates. Although the complexity of this disease and its aggressive behavior make it very difficult to treat, we envision that the study of biomarkers for the early detection and management of GBM will lead us to new accomplishments. Many already-established biomarkers can predict prognosis and treatment response and are of significant value. Given the fact that we have limited, concise results and cannot theorize further, we encourage future studies regarding the matter to help elucidate the complex subject of GBM.

## Data Availability

Not applicable.
